# Functional and Structural Remodeling of Glutamate Synapses in Prefrontal and Frontal Cortex Induced by Behavioral Stress

**DOI:** 10.3389/fpsyt.2015.00060

**Published:** 2015-04-27

**Authors:** Laura Musazzi, Giulia Treccani, Maurizio Popoli

**Affiliations:** ^1^Laboratory of Neuropsychopharmacology and Functional Neurogenomics, Dipartimento di Scienze Farmacologiche e Biomolecolari, Center of Excellence on Neurodegenerative Diseases (CEND), Università degli Studi di Milano, Milano, Italy; ^2^Translational Neuropsychiatry Unit, Department of Clinical Medicine, Aarhus University, Aarhus, Denmark

**Keywords:** mood disorder, glutamate transmission, prefrontal cortex, behavioral stress, neuronal remodeling, working memory

## Abstract

Increasing evidence has shown that the pathophysiology of neuropsychiatric disorders, including mood disorders, is associated with abnormal function and regulation of the glutamatergic system. Consistently, preclinical studies on stress-based animal models of pathology showed that glucocorticoids and stress exert crucial effects on neuronal excitability and function, especially in cortical and limbic areas. In prefrontal and frontal cortex, acute stress was shown to induce enhancement of glutamate release/transmission dependent on activation of corticosterone receptors. Although the mechanisms whereby stress affects glutamate transmission have not yet been fully understood, it was shown that synaptic, non-genomic action of corticosterone is required to increase the readily releasable pool of glutamate vesicles, but is not sufficient to enhance transmission in prefrontal and frontal cortex. Slower, partly genomic mechanisms are probably necessary for the enhancement of glutamate transmission induced by stress. Combined evidence has suggested that the changes in glutamate release and transmission are responsible for the dendritic remodeling and morphological changes induced by stress and it has been argued that sustained alterations of glutamate transmission may play a key role in the long-term structural/functional changes associated with mood disorders in patients. Intriguingly, modifications of the glutamatergic system induced by stress in the prefrontal cortex seem to be biphasic. Indeed, while the fast response to stress suggests an enhancement in the number of excitatory synapses, synaptic transmission and working memory, long-term adaptive changes – including those consequent to chronic stress – induce opposite effects. Better knowledge of the cellular effectors involved in this biphasic effect of stress may be useful to understand the pathophysiology of stress-related disorders, and open new paths for the development of therapeutic approaches.

## Introduction

Starting from the evidence of the antidepressant properties of drugs increasing the availability of monoamines, the pathophysiology of mood and anxiety disorders has been linked for many years to alterations in monoaminergic transmission (1, 2). However, the monoaminergic hypothesis of depression is simplistic and does not explain the delayed pharmacological effect of antidepressants and the high number of non-responding patients. The more recent and well-accepted “neuroplasticity hypothesis” of depression claims that concomitant changes in intracellular signaling, neurotrophic mechanisms, neurogenesis, synaptic function and plasticity, and remodeling of neuronal cells/circuitry are involved in pathophysiology and treatment of mood disorders (3–5). These “neuroplastic” changes are hypothesized to lead to disruption of homeostatic mechanisms, resulting in destabilization and loss of synaptic connections in emotional/cognitive circuitry. The hypothesis is supported by brain-imaging studies in patients with mood and anxiety disorders, showing consistent evidence of volume and connectivity reductions in cortical and limbic brain regions, such as prefrontal cortex (PFC), hippocampus, and amygdala (6–8). Interestingly, since most of the connections between and within these brain areas are glutamatergic, the maladaptive morphological changes described in the brain of depressed subjects are accompanied by alterations in glutamate levels, metabolism, and receptors, suggesting that the dysregulation of glutamate neurotransmission plays an important role in the pathophysiology of neuropsychiatric diseases.

In the present review, we focus on the functional alterations in the glutamate system and in dendritic reorganization and neuronal connectivity in the PFC [a brain region critical for working memory, executive function, and extinction of learning, Ref. (9)], reported in patients with mood disorders and induced by stress in preclinical models. As discussed in the following sections, while acute stress was shown to enhance rapidly the function of the PFC at both cellular and behavioral levels, repeated exposure to stress, as well as long-term effects of some types of acute stressors, bring about atrophy and retraction of dendrites, loss of synapses, reduction of synaptic transmission, and consequent behavioral impairments. Understanding the mechanisms and the molecular effectors involved in this biphasic action of stress is essential to the development of new diagnostic and therapeutic strategies for stress-related neuropsychiatric disorders.

## Clinical Evidence of Glutamatergic and Morphological Dysfunctions in Cortical Areas of Patients with Mood Disorders

### Glutamatergic alterations in brain of depressed patients

Evidence collected from clinical studies showed alterations in the levels of glutamate and of its metabolites in plasma, cerebrospinal fluid, and selected brain areas of patients affected by mood and anxiety disorders.

In particular, increased glutamate levels were measured both in the plasma of depressed patients compared with healthy controls (10) and in post-mortem frontal cortex (FC) and dorsolateral PFC from depressed and bipolar patients, respectively (11). Moreover, region-specific abnormalities in mRNA and protein expression levels of ionotropic glutamate receptor subunits and of associated postsynaptic density proteins were measured in post-mortem studies, suggesting compromised glutamate-mediated synaptic neurotransmission in brain areas from depressed individuals (12, 13).

*In vivo* proton magnetic resonance spectroscopy (1H-MRS) was used to measure glutamate-related metabolites in the brain of depressed patients. Despite some inconsistencies, a large number of studies provided evidence for reduction in Glx levels, a composite measure of glutamate and glutamine, with a minor contribution from GABA and other metabolites, in FC and cingulate regions of depressed subjects in the midst of a current depressive episode, and in non-responders patients (14–23). In contrast, glutamate metabolite measures in the occipital and parietal/occipital regions have been found to be elevated in medication-free major depression patients (4, 19, 24). Apart from technical limitations of 1H-MRS studies, these findings strongly suggest that abnormalities in glutamate/glutamine/GABA cycling are involved in the pathophysiology of mood and anxiety disorders.

### Volumetric and morphological changes in the cortex of depressed patients

A large number of clinical neuroimaging studies of depressed patients have consistently shown regional volumetric changes in brain areas where glutamate neurons and synapses predominate (6–8, 25). In particular, significant volumetric reduction has been found for cortical areas, where reduced gray matter volume in the anterior cingulate cortex, PFC, and lateral and medial orbitofrontal cortices were reported (26–28). Interestingly, reduced volume of the caudal anterior cingulate cortex and altered white matter integrity in the body of the corpus callosum were also recently reported in untreated patients with first episode of major depression (29).

In a separate study, decreased PFC gray matter volume and density were measured in depressed patients compared to both healthy controls subjects and remitted major depression disorder subjects (30). Interestingly, morphometric studies on PFC of depressed subjects showed smaller size of neuronal bodies and reduced neuronal and glial densities, thus suggesting that volumetric changes are at least in part dependent on neuronal abnormalities (25, 31–33). In line with this hypothesis, decreased expression of synapse-related genes and loss of synapses have been recently described in PFC of patients with mood disorders, confirming that synaptic dysfunction contributes to the volumetric changes observed in depressed patients (34).

Indeed, although the reasons for morphological changes in brain areas of depressed subjects have not yet been fully understood, it has been proposed that atrophy and remodeling of dendrites and reduction of synapses are major factors (4–6, 35). In particular, since the brain areas where volumetric changes were reported are prevalently glutamatergic (6–8, 25), it is conceivable that glutamate synapses are particularly affected in the pathology. The evidence for this hypothesis mainly comes from preclinical stress-based models of mood and anxiety disorders. Indeed, the morphological changes induced in brain areas of chronically stressed animals were found to be accompanied by dysfunctional glutamatergic synaptic plasticity and transmission (see below).

## Glutamatergic and Morphological Dysfunctions in Cortical Areas of Preclinical Stress-Based Models of Mood and Anxiety Disorders

Since behavioral stress is recognized as a major predisposing and triggering factor for mood and anxiety disorders in humans, the high majority of rodent models of depression are based on the exposure to standardized acute and chronic stress protocols (36–38). In this section, we report the structural/functional changes induced by several protocols of chronic and acute stress in PFC glutamate synapses and circuitry.

### Morphological changes and PFC-dependent cognitive impairment induced by chronic stress

It has been widely documented that, after prolonged stress, pyramidal neurons in medial PFC undergo dendritic atrophy, reduction of synapses number and volumetric reductions, resembling those observed in patients with mood and anxiety disorders (Table 1). In particular, different protocols of chronic stress were reported to induce dendritic remodeling of pyramidal neurons in layers II/III, where reductions in total apical dendritic length, arborization, and spine density were consistently reported (39–63). Recent studies also demonstrated a significant retraction after chronic stress of layer V pyramidal neurons apical dendrites within distal cortical layers (64–66).

**Table 1:** **Effect of chronic stress on neuronal remodeling in the prefrontal cortex: animal studies**.

Stress	Morphological changes	Reference
Restraint stress (21 days)	Reduction in the number and length of apical dendritic branches in distal and higher-order branches in layer II/III pyramidal neurons	(39)
Social isolation (8 weeks)	Reductions in dendritic spine density in layer III pyramidal neurons	(40)
Restraint stress (21 days)	Reduction in the total length and branch numbers of apical dendrites in layer II/III pyramidal neurons of infralimbic and prelimbic cortices	(41)
Restraint stress (7 days)	Atrophy of distal branches and sparing of proximal branches in layer II–III pyramidal neurons	(42)
Restraint stress (3 and 6 weeks)	Reduction in total apical dendritic length in layer II/III pyramidal neurons	(43)
Forced swim (3 days)	Retraction of terminal branches of apical, but not basilar, dendrites in infralimbic cortex pyramidal neurons	(44)
Restraint stress (21 days)	Retraction of apical dendritic arbors in in layer II/III pyramidal neurons	(45)
Chronic noise stress (30 days)	Reduction in the number of apical dendrites in layer II/III pyramidal neurons	(46)
Restraint stress (21 days)	Reduction in the total length and branch numbers of apical dendrites and of axospinous synapses number in layer II/III pyramidal neurons of prelimbic cortices	(47)
Prenatal stress (7 days) followed by chronic mild stress (3 weeks)	Reduction in spine densities, particularly on spines of the mushroom type in medial PFC	(48)
Restraint stress (14 days)	Reduction in the total length of apical dendrites in prelimbic cortex pyramidal neurons	(49, 50)
Restraint stress (21 days)	Decrease in dendritic spine volume and surface area, mainly in the distal portion of apical dendritic fields; reduction in large spines and increase in small spines	(51)
Chronic unpredictable stress (21 days)	Volumetric and dendritic atrophy in layer II/III pyramidal neurons of infralimbic and prelimbic cortices	(52)
Restraint stress (7 days)	Reduction in the number and length of apical dendritic branches in layer II/III pyramidal neurons of infralimbic and prelimbic cortices (selectively in male and not female mice)	(53)
Restraint stress (21 days)	Reduction in apical dendritic length and in apical dendritic branch intersections in layer II/III pyramidal neurons	(54)
Restraint stress (10 days)	Retraction of apical dendrites in infralimbic cortex pyramidal neurons	(55)
Restraint stress (21 days)	Reduction in apical dendritic length and in apical dendritic branch intersections in layer II/III pyramidal neurons of prelimbic cortex	(56)
Restraint stress (7 days)	Retraction of apical dendrites in layer II/III pyramidal neurons	(57)
Restraint stress (21 days)	Reduction in the number and length of apical dendritic branches in prelimbic cortex pyramidal neurons	(58)
Prenatal stress (7 days)	Reduction in dendritic complexity in prelimbic cortex pyramidal neurons	(59)
Isolation (8–9 weeks)	Reduction in dendritic complexity, spine density, and elongated terminal branches in layer II/III pyramidal neurons	(60)
Early life stress (maternal separation, 14 days)	Atrophy of basal dendritic tree and reduced spine density on both apical and basal dendrites in layer II/III pyramidal neurons	(61)
Prenatal stress (7 days)	Decrease in the apical dendritic length of pyramidal neurons in the orbitofrontal cortex at postnatal day 14	(62)
Prenatal stress (7 days)	Apical dendrite arbor simplification in layer III pyramidal neurons	(63)
Restraint stress (3 or 7 days)	Atrophy of distal apical dendritic in layer V pyramidal neurons	(64)
Restraint stress (21 days)	Atrophy of apical dendritic tree and reduced spine density in layer V pyramidal neurons of infralimbic cortex	(65)
Chronic unpredictable stress (21 days)	Reduction in spine density in both distal and proximal dendrites in layer V pyramidal neurons	(66)

Interestingly, a number of convergent studies reported that the reduction of spine densities, apical dendritic length, and branch points in medial PFC layer II/III pyramidal neurons, induced by chronic stress in prenatal, early life or adult life, is accompanied by behavioral impairments in tests for emotional/cognitive behavior (52, 56, 58, 60–62, 66). Chronic restraint stress (21 days) was shown to selectively impair attentional set-shifting task (a function mediated by medial PFC), together with retraction of apical dendritic arbors in anterior cingulate cortex and extension in lateral orbitofrontal cortex (45), and to worsen working memory in the spatial delayed alternation T-maze task, in concomitance with atrophy of apical dendrites in pyramidal neurons from layer II/III of prelimbic cortex (54). It was also shown that the retraction of apical dendrites of pyramidal neurons induced in PFC by repeated stress is accompanied by alterations in fear conditioning and extinction (44).

Together, all these lines of evidence are clearly in favor of a correlation between chronic stress, dendritic remodeling, and impaired PFC-dependent cognitive performance.

Although the mechanisms underlying the effects of chronic stress on PFC are far from being fully elucidated, it was consistently reported that chronic systemic injections of the stress hormone corticosterone are able to reproduce, at least in part, the structural and functional changes induced by stress in this brain area. Chronic high-dose systemic injections of corticosterone were shown to cause significant reduction of spines in PFC pyramidal neurons of layer V and deficits in memory retention (67), and to induce a significant redistribution of apical dendrites in layers II/III, with an increase in the number of proximal dendrites and a reduction of distal dendrites (68). Repeated corticosterone injections within infralimbic and prelimbic medial PFC were also found to impair working memory and to improve memory consolidation, through a glucocorticoid receptor-dependent mechanism (69). On the other hand, repeated corticotropin-releasing factor infusion directly into the medial PFC increased general anxiety, but did not affect cue-conditioned fear 10 days post infusion (70). Moreover, some studies also reported an involvement in dendritic remodeling of a number of intracellular signaling mediators and receptors, including protein kinase C (54), glucocorticoid receptor (71), NMDA receptors (57), AMPA receptors, postsynaptic density protein 95, α calcium/calmodulin-dependent protein kinase II (61), estrogen (63, 72), glutamate decarboxylase enzyme 64, NCAM, synaptophysin and GABA(A) α 1 (73), catecholamines (64, 74), and cannabinoid CB1 receptor (56).

### Alterations in synaptic transmission and related molecular mechanisms induced by chronic stress in the prefrontal cortex

Together with morphological and behavioral changes, a number of studies analyzed the effects of chronic stress on synaptic function, i.e., presynaptic glutamate release and function/membrane insertion of postsynaptic glutamate receptors.

An early paper correlated electrophysiological and morphological changes in medial PFC layer V pyramidal neurons, using a combination of whole-cell recording and two-photon imaging in rat medial PFC slices (64). The authors clearly showed that repeated mild restraint stress, together with a decrease in distal apical dendritic branch length and spine density, induced deficits in apically targeted excitatory postsynaptic currents (EPSCs), involving corticosterone-dependent mechanisms. Similarly, the reduction of large mushroom spines of layer V pyramidal neurons measured after chronic unpredictable stress was found to be accompanied by a reduction in the amplitude and frequency of serotonin- and hypocretin-induced EPSCs (66). More recently, a reduction of both AMPA and NMDA receptor-dependent synaptic responses and spontaneous action potential firing in pyramidal PFC cells were reported after 5–7 days of restraint or unpredictable stress in young rats, in association with ubiquitin/proteasome-mediated degradation of selective glutamate receptor subunits (74, 75).

Moreover, sex differences were shown in PFC excitatory transmission, glutamate receptor surface expression, and behavioral response after repeated restraint stress (72). Indeed, chronic stress-induced memory impairment together with decreased AMPA and NMDA receptors surface expression, receptor-dependent EPSCs, and miniature EPSCs, in medial PFC of male rats selectively, with no effect in female rats. Looking for potential mechanisms underlying the contrasting effects of repeated stress on PFC functions in female vs. male animals, the authors demonstrated that estrogen both protects against the detrimental effects of repeated stress in females, and prevents the stress-induced impairments when administered to males. This suggests that the stress hormone corticosterone and estrogen interact in the modulation of excitatory synaptic transmission in PFC neurons, leading to a fine tuning of functional plasticity within this brain area. In this context, it is interesting to notice that local brain synthesis of estrogen from endogenous cholesterol, through the action of neuronal aromatases, could play a role in the modulation of neurotransmission in response to repeated stress. Indeed, it was shown that the inhibition of aromatase in female rats resulted in the loss of protection against neural and behavioral consequences of chronic stress, thus suggesting that central estrogen production is necessary for the protective action of estrogen (72).

In addition to neuronal structure, chronic stress was also reported to induce impairments in synaptic plasticity (i.e., long-term potentiation, LTP) in medial PFC (61, 65). The decrease of spine density in both apical and basal dendrites and the atrophy of the basal dendritic tree in medial PFC layer II/III pyramidal neurons, induced by chronic early life stress (maternal separation) in young rats, were found to be accompanied by attenuation of LTP and changes in the expression of proteins involved in LTP, including AMPA receptor subunits (61). Moreover, in a different study, chronic restraint stress was found to inhibit D1 receptor-dependent LTP, while post-stress recovery fully reversed the impairments in catecholaminergic-mediated synaptic plasticity, suggesting that recovery may be related with circuitry reestablishment (65).

These studies strongly suggest that the structural remodeling induced by chronic stress in PFC is accompanied by dysfunctions in neurotransmission and plasticity.

Regarding the effects of chronic stress on glutamate levels and presynaptic release, less information is available. Early evidence was provided by microdialysis studies, which found selective changes in the adaptation of extracellular glutamate in hippocampus and PFC after application of a few consecutive stressors (76). In a more recent study, it was found that glutamate release induced by BDNF in slices of the PFC was attenuated in rats subjected to chronic restraint stress, coupled with anxious/depressive phenotype and down-regulation of glucocorticoid receptors (71). Moreover, reduction in the levels of glutamate, glutamine, *N*-acetyl aspartate, and taurine was reported in the PFC of social defeated mice (77).

Together, these studies strongly suggest that dendritic atrophy and volumetric reduction induced by chronic stress in PFC are related with changes in glutamate transmission and plasticity and, ultimately, may induce severe behavioral deficits.

### Acute stress increases glutamate transmission and release in prefrontal cortex

Although the effects of chronic stress on glutamate release and transmission remain largely unknown, compelling preclinical studies have clearly shown that acute stress and glucocorticoids can deeply affect glutamatergic neurotransmission in the PFC, inducing changes in glutamate release, glutamate receptors, and glutamate clearance and metabolism [as a review, see Ref. (78, 79)].

A study measuring changes in glutamate release in PFC, by using enzyme-based microelectrode arrays coupled to amperometric recording techniques, showed significant transient increase of extracellular glutamate levels during tail pinch stress, which was completely blocked by local application of tetrodotoxin, thus suggesting increased exocytotic release of glutamate (80).

In rat PFC and FC, we have shown that acute stress rapidly enhances glutamate release and transmission, an effect mediated by corticosterone receptors. We applied one single 40 min session of inescapable footshock (FS)-stress to rats, and then purified synaptic terminals (synaptosomes) from PFC and FC with Percoll gradients (81). Basal and depolarization-evoked glutamate release was measured by using the technique of isolated synaptosomes in superfusion, a method allowing to measure the exocytotic release of neurotransmitters, preventing or limiting reuptake by neurotransmitter transporters, or synaptic receptors activation (78, 82). Acute FS-stress-induced rapid enhancement of depolarization-evoked glutamate (not GABA) overflow in PFC and FC, by increasing corticosterone levels, stimulation of corticosterone receptors, and rapid accumulation of presynaptic SNARE protein complexes, which mediate vesicle fusion (81). Enhancement of glutamate release was confirmed by reduction of paired-pulse facilitation and its calcium-dependence in PFC of stressed rats. It was also shown that chronic antidepressants prevent the enhancement of glutamate release, with a mechanism downstream of corticosterone rise.

More recently, the synaptic effects of acute stress and corticosterone in PFC and FC were dissected, showing that the increase of corticosterone induced by FS-stress is responsible for a rapid (non-genomic) enhancement of trafficking of glutamate synaptic vesicles into the so-called readily releasable pool (RRP), through the activation of synaptic glucocorticoid/mineralocorticoid receptors (83). Indeed, both acute stress and brief *in vitro* application of corticosterone to purified PFC and FC synaptosomes were shown to increase the RRP size of glutamate vesicles (assessed in synaptosomes superfused with hypertonic sucrose). In line with this evidence, FS-stress increased the number of synaptic vesicles docked onto presynaptic membranes of excitatory perforated synapses, measured with electron microscopy stereology in medial PFC (83, 84). Similarly, by using total internal reflection fluorescence microscopy, we showed that application *in vitro* of corticosterone to synaptosomes for up to 10 min rapidly increased trafficking of FM1-43 labeled synaptic vesicles toward the presynaptic membrane. The increase of vesicle mobilization and RRP induced by both acute stress and *in vitro* corticosterone application was demonstrated to be dependent on the activation of synaptic corticosterone receptors and downstream increase of site 1 (Ser9) synapsin I phosphorylation in presynaptic membranes (83). Indeed, despite the molecular mechanism involved in corticosterone-induced increase of vesicle mobilization is far to be fully elucidated, the phosphorylation of synaptic membrane-located synapsin I at site 1 was found to be necessary for the enhancement of RRP.

**Figure 1:**
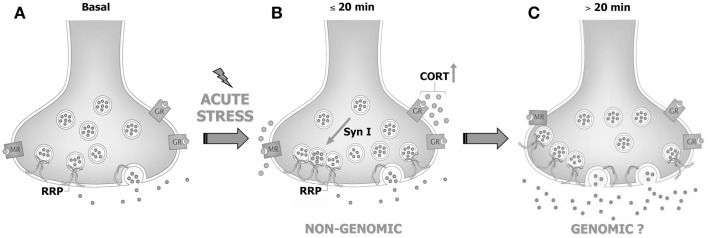
**Acute stress and corticosterone rapidly increase the readily releasable pool of glutamate vesicles in prefrontal and frontal cortex synaptic terminals through non-genomic mechanisms**. **(A)** PFC glutamate synaptic terminal in basal conditions, showing the readily releasable pool (RRP) of vesicles anchored to the membrane and ready for release and membrane-located mineralocorticoid and glucocorticoid receptors (MR and GR, respectively). **(B)** The rise of corticosterone (CORT) induced by acute stress causes a rapid increase of vesicle trafficking into the RRP. This requires binding of corticosterone to synaptic MR/GR and non-genomic mechanisms involving phosphorylation of the presynaptic protein synapsin I. The increase of RRP size non-genomically induced by corticosterone primes the terminal but is not sufficient for the enhancement of glutamate release and transmission induced by acute stress. **(C)** We speculate that slower (>20 min), likely genomic effects are required to promote the enhancement of glutamate release and transmission induced by acute stress. See text for details. Adapted from Treccani et al. (86).

However, while corticosterone rapidly primes synapses for enhanced release, it does not likewise rapidly enhance glutamate release and transmission. Indeed, acute application of corticosterone failed to reproduce the stress-induced increase of depolarization-evoked release of glutamate in purified PFC and FC synaptosomes, and to induce any change in intracellular EPSCs amplitude or paired-pulse ratio in acute medial PFC slices (83, 85), suggesting that the synaptic non-genomic action of corticosterone is necessary but not sufficient to enhance glutamate release and transmission [see Ref. (86); Figure 1]. In line with this hypothesis, it was consistently reported that both acute stress and corticosterone induce delayed and long-lasting potentiation of glutamate transmission in the PFC (see below).

### The increase of glutamate transmission and release in prefrontal cortex is mediated by slower, likely genomic, corticosterone effect

Acute stressors of diverse types, including acute forced swim, acute restraint, and elevated-platform stress, as well as acute corticosterone treatment of rats, were shown to induce in PFC pyramidal neurons a significant long-lasting potentiation, starting 1 h after stress and sustained for up to 24 h after cessation of stress, of glutamatergic transmission and an increase of surface NMDA and AMPA receptor subunits level, through the activation of glucocorticoid receptors (87). This was the first evidence showing that corticosterone is necessary and sufficient for increasing glutamate transmission in PFC, and also suggested that the potentiation of glutamate transmission in this brain area involves delayed mechanisms (no significant effect measured until 1 h after corticosterone elevation). Similarly, acute *in vitro* treatment of both PFC neuronal cultures and acute PFC slices was shown to induce synaptic potentiation at least 1 h and up to 24 h after corticosterone application (88, 89). These studies also showed that the long-lasting potentiation of glutamatergic transmission in PFC pyramidal neurons is likely caused by the increase in the delivery of NMDA and AMPA receptors to the synaptic membrane, in turn dependent on activation of glucocorticoid receptors and glucocorticoid-inducible kinase/Rab4 signaling (88).

Although these studies suggest that the enhancement of glutamate release and transmission induced by acute stress in PFC is dependent on the transcriptional activation of immediate early genes, other studies also suggested non-genomic, synaptic effects of corticosterone. Indeed, it was shown that both acute stress and incubation of medial PFC slices with corticosterone induces rapid changes in neurotransmission, dependent on local synthesis of endocannabinoids, thus suggesting that some of the short-term effects of corticosterone may be partly mediated by the local release of other mediators (56). In particular, the activation of endocannabinoid signaling induced by corticosterone, inhibiting GABA release onto layer V pyramidal neurons in the prelimbic cortex, contributes to the long-negative feedback loop to inhibit corticosterone secretion following cessation of stress.

### Behavioral and morphological changes induced by acute stress

Although chronic stress has been widely shown to induce profound structural remodeling of medial PFC and related behavioral alterations (see Morphological Changes and PFC-Dependent Cognitive Impairment Induced by Chronic Stress), less is known about the effects of acute stress on PFC synaptic plasticity and memory. Neurons in cortical and limbic areas are highly plastic and undergo rapid activity-dependent morphological transformations, including modulation of neuronal excitability and connectivity (90, 91). However, only a few recent studies focused on the changes in structural remodeling and in PFC-dependent behavioral performance induced by a single stressful event. Interestingly, as described in detail below, the changes in synaptic plasticity and memory seem to be time-dependent and biphasic, inducing a general enhancement of transmission, plasticity, and performance in the first hours after stress, followed by negative effects on learning, memory, and their neural underpinnings, in the subsequent hours and days.

In a recent study, we have shown that both acute FS-stress and restraint stress induce marked effects on synaptic plasticity, increasing the total number of asymmetric (i.e., excitatory) non-perforated synapses in pyramidal neurons of prelimbic PFC layers II/III (84). FS-stress, a stress protocol inducing significantly higher levels of corticosterone compared with restraint stress, also increases the number of axo-shaft synapses, directly located on dendritic shafts. Interestingly, these changes were partially blocked by chronic antidepressant pretreatment, as previously show for glutamate release and transmission (81), thus providing a parallel between the modulation of excitatory transmission induced by antidepressants and changes in structural remodeling. Moreover, our findings provide a first evidence that activity-dependent synaptogenesis of small synapses can occur *ex novo* in PFC, as early as 40 min after a severe stressful event, confirming the remarkable dynamics of synapse structure in response to stressful events in this brain area.

In line with these data, the plasticity enhancing effect induced by acute forced swim stress and elevated-platform stress in PFC glutamate transmission was associated with enhanced working memory in the T-maze delayed alternation task when examined 4 h or 1 day after stress, but not 2 days after stress (87). The enhancement of working memory, as for the increase in glutamate transmission, was shown to be dependent on glucocorticoid receptors and on the activation of glucocorticoid receptor and glucocorticoid-inducible kinase dependent mechanisms (88).

Finally, in a separate study, acute forced swim stress was found to induce a significant retraction of apical (not basal) branches, measured 3 days after stress (44). However, the remodeling of dendritic arbor was found to be selectively located in infralimbic, but not prelimbic PFC, suggesting that pyramidal neurons within infralimbic PFC are highly sensitive to forced swim stress.

## Conclusion

Compelling evidence strongly suggests that long-term changes in brain areas and circuits, mediating complex cognitive and emotional behaviors, represent the biological underpinnings of mood and anxiety disorders. Stressful life events deeply affect brain function and, especially when stress exposure is intense, chronic, uncontrollable, or overwhelming, it represents a major risk factor for many diseases, including neuropsychiatric disorders.

The stress response is a complex physiological process involving hormonal, neurochemical, and metabolic mechanisms. Indeed, although PFC was consistently reported to be a brain area particularly sensitive to stress, it is important to notice that stress induces changes in connectivity and plasticity, not only within PFC but also in other areas, such as hippocampus and amygdala, and between different areas [as a review, see Ref. (92)]. Intriguingly, the effects of stress seem to be region-specific. Indeed, as for PFC, in hippocampus acute stress was shown to deeply affect glutamatergic transmission in preclinical models. However, differently from PFC, in hippocampus the activation of synaptic corticosterone receptors, and particularly mineralocorticoid receptor, was found to be sufficient to induce rapid enhancement of glutamate release and synaptic transmission, through completely non-genomic mechanisms (93, 94). On the other hand, opposite effects were noticed in amygdala, where the enhancement of glutamate transmission induced by stress in rodents is accompanied by increased dendritic complexity (95, 96). Moreover, PFC, hippocampus, and amygdala also critically participate in orchestrating the hypothalamic–pituitary–adrenal (HPA) axis response to stress, thus modulating the physiological stress response (97). Understanding how these stress-related networks operate could be helpful in uncovering pathways mediating pathological stress-related conditions.

**Figure 2:**
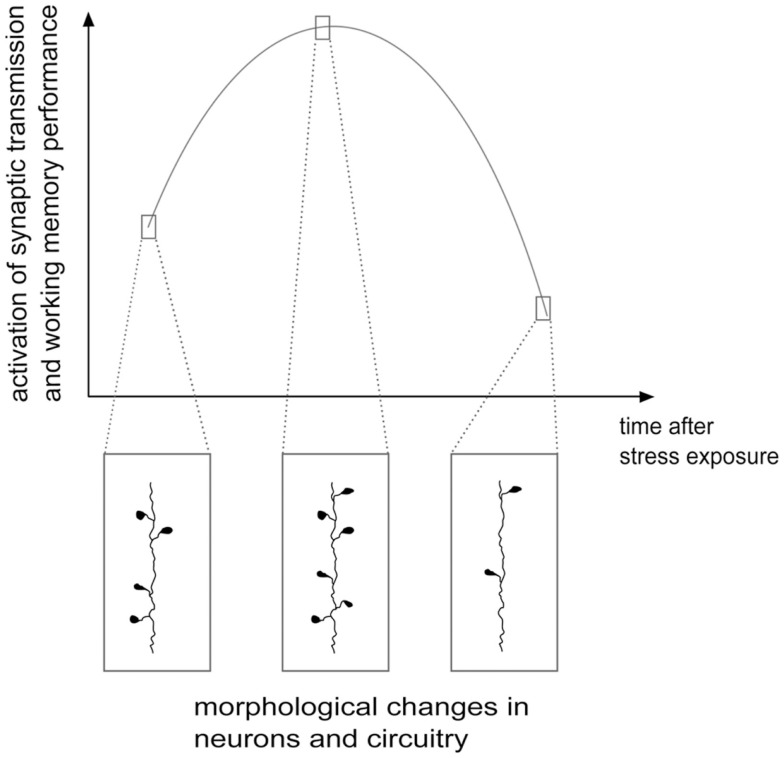
**Hypothetical scheme of structural/functional changes induced by stress in the glutamate system: a biphasic process**. Stress and corticosterone were shown to induce enhancement of excitatory synaptic transmission and increase in the number of spines and synapses, often accompanied by cognitive enhancement, in the first several minutes and hours. Later on, at least 24 h after application of the stressor, a phase of inhibition follows, with reduction of synaptic transmission, dendritic atrophy and remodeling, loss of spines and synapses and negative effects on cognitive functions. See text for details. Adapted from Musazzi et al. (98).

As shown above, although the evidence is far from conclusive, the acute and delayed (e.g., after repeated or chronic stress) outcome on structural and functional features of the glutamate system could be different and often opposite, at least in the PFC. On one side, stressful events rapidly enhance glutamate release and excitatory transmission and may facilitate plasticity and PFC-dependent behavior, while chronic stress and long-term effects of some acute stressors induce a reduction of excitatory transmission, atrophy/remodeling of dendrites and loss of synapses, accompanied by behavioral impairment. Therefore, the structural and functional changes in excitatory circuitry may follow a biphasic process, during which, at some unknown points, the stress response turns from increased excitatory activation into its opposite [Figure 2; see Ref. (78, 98, 99) for a discussion]. Thus, upon severe acute stressful stimulation, HPA axis response is triggered, as shown by elevated glucocorticoids levels, in concert with strong induction of PFC function. This overall marked potentiation, most likely promoted to face the initial threat and facilitate induction of the memory of the stressor, may subsequently produce progressive exhaustion of the system, which in turn results into deep impairment of mPFC-mediated function and neuroarchitecture. Therefore, the biphasic effect of stress on synaptic transmission, morphology, and behavioral performance may be considered a compensatory physiologically adaptive response to environmental stressors. However, if the stress response is inadequate or dysregulated, because the stressor is prolonged or overcomes the coping capability of the system, the structural/functional changes may disrupt homeostasis, thus increasing the risk to develop a stress-related pathology (35, 99).

Understanding what regulates the turning point between a physiological adaptive stress response and the beginning of maladaptive remodeling will be crucial in the study of pathophysiology of neuropsychiatric stress-related disorders. In this context, considering that a high majority of individuals, although exposed to traumatic experiences during lifespan, do not develop stress-related neuropsychiatric disorders, a better knowledge of the molecular/cellular effectors regulating the individual susceptibility to stress could be of great help to mitigate the detrimental effects of external threats and/or to increase the resilience of individuals to stressful events (100).

## Author Contributions

All authors contributed to the design and content of the manuscript as well as the first draft of the manuscript and all subsequent revisions. All authors have approved the final version of the manuscript.

## Conflict of Interest Statement

The authors declare that the research was conducted in the absence of any commercial or financial relationships that could be construed as a potential conflict of interest.
